# A Novel Staining Method for Detection of Brain Perivascular Injuries Induced by Nanoparticle: Periodic Acid-Schiff and Immunohistochemical Double-Staining

**DOI:** 10.3389/ftox.2022.825984

**Published:** 2022-03-21

**Authors:** Atsuto Onoda, Shin Hagiwara, Natsuko Kubota, Shinya Yanagita, Ken Takeda, Masakazu Umezawa

**Affiliations:** ^1^ The Center for Environmental Health Science for the Next Generation, Research Institute for Science and Technology, Tokyo University of Science, Noda, Japan; ^2^ Faculty of Pharmaceutical Sciences, Tokyo University of Science, Noda, Japan; ^3^ Faculty of Pharmaceutical Sciences, Sanyo-Onoda City University, Sanyo-Onoda, Japan; ^4^ Graduate School of Human Health Sciences, Tokyo Metropolitan University, Hachioji, Japan; ^5^ Institute of Arts and Sciences, Tokyo University of Science, Noda, Japan; ^6^ Department of Materials Science and Technology, Faculty of Advanced Engineering, Tokyo University of Science, Tokyo, Japan

**Keywords:** developmental neurotoxicity, histopathology, perivascular macrophage, astrocyte, nanoparticle, periodic acid-Schiff, immunohistochemistry, central nervous system

## Abstract

**Background:** To protect developing brain from any unfavorable effects, it is necessary to construct experimental techniques that can sensitively detect and evaluate developmental toxicity. We have previously shown that brain perivascular tissues, especially perivascular macrophages (PVMs), respond sensitively even to weak stimuli by foreign toxicants such as low-dose exposure to nanoparticle. This paper shows the protocol of a novel staining method that enables easy detection and rapid evaluation of brain perivascular abnormalities.

**Methods:** As weak stimulus, low-dose of carbon black nanoparticle (95 μg/kg) or titanium dioxide nanoparticle (100 μg/kg) was intranasally administered to pregnant mice at gestational days 5 and 9. The offspring brains were used to confirm the properties of PVMs and to find suitable protocols for the detection and evaluation of the mild denaturation of PVMs. Furthermore, various procedures of novel combinational double staining including periodic acid-Schiff (PAS) staining and immunohistochemistry were examined. In addition, we checked the alterations in neurotransmitter levels and the behaviors of the offspring.

**Results and discussion:** Maternal exposure to low-dose of nanoparticle at levels where no significant effects on the brain were observed, such as abnormal behavior, alteration of neurotransmitter levels, or microglial activation, resulted in mild denaturation of the PVMs, which was captured by PAS staining. However, it was difficult to detect and determine slight histopathological alterations. Therefore, we established PAS-immunohistochemical double-staining method for the brain. This double staining method enabled easy detection and rapid evaluation of brain perivascular abnormalities and the relationship between PVMs and the surrounding cells. In addition, this double staining allows evaluation of the histopathological denaturation of the PVMs and the associated abnormalities in the surrounding tissues in the same section.

**Conclusion:** The slight responses of brain perivascular tissues, such as mild denaturation of PVMs, were sensitively and easily determined by the PAS-immunohistochemical double-staining method. This double staining method is a powerful tool to assess brain perivascular injuries including PVM denaturation and the relationship between the expression of various molecules and the morphology of PVMs. We propose that the observation of the tissue around brain blood vessels using the double staining provides potential endpoints to evaluate developmental neurotoxicity.

## Introduction

Developmental toxicology is a struggle with detection sensitivity. Developing fetuses and newborns are sensitive to external stimuli because their toxicokinetics and toxicodynamics are quite different from those of adults (Saunders and Dziegielewska, 2007). Their development can be disrupted by slight adverse impacts during the perinatal period, even if it is harmless to adults (Grandjean and Landrigan, 2006; Miodovnik, 2011; Grandjean and Landrigan, 2014). To prevent unfavorable abnormal development, it is necessary to construct an experimental system that can sensitively detect and evaluate the developmental toxicity of various unknown and novel substances; to achieve this goal, experimental techniques that can capture and evaluate slight biological reactions at an early stage before they appear as phenotypes are essential.

We have previously investigated the adverse effects of nanoparticles on the developing brain. Prior studies using animal models have demonstrated that maternal exposure to low-dose of nanoparticle, which can exist in the real world, induced histopathological injuries around the brain blood vessels of the offspring (Onoda et al., 2017a; Onoda et al., 2017b); in particular, brain perivascular macrophages (PVMs) respond sensitively to exposure (Onoda et al., 2014; Onoda et al., 2020). PVMs have unique localization and function, unlike ordinary macrophages. They are resident macrophages located at brain-perivascular space (the interface between the brain parenchyma tissues and blood vessels) and directly contact to the cerebrospinal fluid. (Lee et al., 2021). PVMs play an important role in preventing foreign substances, such as viruses, from invading the brain parenchyma through blood vessels (Abreu et al., 2019a; Abreu et al., 2019b; Bohannon et al., 2020). Furthermore, they function to keep the brain perivascular space clean by phagocytosing waste and unnecessary substances that are discharged by the brain parenchyma into the perivascular space (Faraco et al., 2017; Yang et al., 2019). Unlike microglia, which only exhibit phagocytosis when activated, PVMs always express scavenger receptors and exert a high phagocytic capacity (Guillemin and Brew, 2004; Lee et al., 2021). Furthermore, PVMs act as antigen presenting and immune surveillant cells and regulate blood-brain barrier permeability (Yang et al., 2019). In the early stages of various neurodegenerative diseases and brain disorders, functional and histopathological abnormalities of PVMs and surrounding astrocytes have been observed and associated with exacerbation of diseases (Maragakis and Rothstein, 2006; Park et al., 2017; Sweeney et al., 2018; Bogale et al., 2021). Because PVMs, located at the front line of brain defense and constantly protect the brain parenchyma, are the first cells to respond to foreign substances circulating in blood and cerebrospinal fluid, they may be a useful end-point to investigate developmental toxicity following exposure to external toxicants and pollutants.

Histopathological analysis using various staining methods is an effective approach to detect and evaluate brain perivascular injuries including PVM denaturation (Lee et al., 2021). In contrast, methods using homogenized tissues make it difficult to analyze only PVMs because they are a minority in the brain cell population (Guillemin and Brew, 2004). Thus, it is necessary to use histopathological analysis, including immunohistochemistry and other staining techniques, to evaluate the morphology of PVMs. In addition, histopathological analysis normally requires advanced skills to determine cell morphology and pathological changes under various types of microscopes. Identification of slight changes in the initial lesion site in the developing brain is particularly difficult. In the case of PVMs, more than sufficient training is required to detect the cells and to determine their histopathological changes. Since the typical histopathological changes in PVMs mainly occur in intracellular granules (Mato et al., 2002; Mato et al., 2009), the ability to observe their inconspicuous changes in the order of 1 μm is required to investigate their denaturation. Therefore, we have established a novel staining method to easily detect and rapidly evaluate the denaturation of PVMs associated with brain perivascular injuries.

## Materials and Methods

### Preparation of Nanoparticle Suspension

The carbon black nanoparticle (CB-NP) suspension was prepared according to previously reported methods (Shimizu et al., 2014; El-Sayed et al., 2015; Onoda et al., 2021). CB-NP (Printex 90) used were obtained from Degussa Ltd., Frankfurt, Germany. The primary particle diameter of Printex 90 was approximately 14 nm, and its surface area was 295–338 m^2^/g. The CB-NP contained >99% carbon, 0.82 weight percent (wt%) nitrogen, 0.01  wt% hydrogen, and <1 wt% organic and inorganic impurities. The CB-NP was suspended in ultrapure water at a concentration of 5 mg/ml, and the suspension was ultrasonicated for 30 min and immediately filtered through a 450 nm filter (S-2504, Kurabo Co., Ltd., Osaka, Japan) before exposure to remove agglomeration. The particles in the filtered suspension were characterized by dynamic light scattering (NANO-ZS, Sysmex Co., Kobe, Hyogo, Japan) using the Rayleigh-Debye equation and field-emission scanning electron microscopy (FE-SEM) (JSM-6500F, JEOL Ltd., Tokyo, Japan) on a silicon wafer. The mode value estimated by dynamic light scattering was 68 nm (Shimizu et al., 2014). In addition, the characteristic diameter determined by electron microscopy was approximately 50–500 nm (Shimizu et al., 2014). The CB-NP concentration in the suspension was calculated as 95 μg/ml by the peak area of the carbon signal (2.77 keV) obtained using an FE-SEM (JSM-6500F) with an attached energy-dispersive X-ray analyzer (Onoda et al., 2014).

The titanium dioxide nanoparticle (TiO_2_-NP) suspension was prepared according to previously reported methods (Tachibana et al., 2021). TiO_2_-NP suspension (rutile: anatase = 20: 80) was purchased from Sigma-Aldrich Co. (700347-25G, St. Louis, MO, United States). The secondary particle diameter of the TiO_2_-NP was less than 150 nm, and the primary particle diameter of the starting nanopowder was 21 nm. The TiO_2_-NP suspension was filtered through a 450 nm filter (Millex®-HV; Cat. No. SLHU033RS; Merck Millipore Ltd., Burlington, MA, United States) to remove the agglomeration. After filtration, the TiO_2_-NP suspension was diluted to 100 μg/ml with distilled water. The size distribution of the hydrodynamic diameter of TiO_2_-NP in the suspension was quantified by dynamic light scattering (Zetasizer Nano ZS, Malvern Instruments Ltd., Malvern, United Kingdom) using the Rayleigh-Debye equation, and the estimated mode value was 79 nm. Transmission electron microscopy (TEM) (JEM 1200EXII, JEOL Ltd., Akishima, Tokyo, Japan) on a collodion-coated 200 Cu mesh (Cat. No. 6511; Nisshin EM, Tokyo, Japan) showed small agglomerates with a characteristic diameter of approximately 25–100 nm (Tachibana et al., 2021).

Ultra-pure water without any nanoparticles was prepared for the control group. In water, no particulate signals were detected by dynamic light scattering and electron microscopy.

### Animals and Exposure

Pregnant Institute of Cancer Research (ICR) mice (11-week-old) at gestational day 3 were obtained from CLEA Japan, Inc. (Tokyo, Japan). The mice were randomly assigned to control (*n* = 8), CB-NP (*n* = 8), and TiO_2_-NP (*n* = 5) groups. The mice in each group were housed in cages (one pregnant mouse/cage) under controlled conditions (temperature: 22 ± 1°C, humidity: 50 ± 5%) with a 12-h light/12-h dark cycle and ad libitum access to food and water. Pregnant mice were treated with intranasal instillation (1 ml/kg body weight) of ultra-pure water (control group), CB-NP suspension (CB-NP group), or TiO_2_-NP suspension (TiO_2_-NP group) under anesthesia with isoflurane on gestational days 5 and 9. After birth, the number of pups per dam was adjusted to 10 on postnatal day 1. All experiments were performed in accordance with the Animal Research: Reporting *In Vivo* Experiments guidelines for the care and use of laboratory animals (Kilkenny et al., 2010) and were approved by the Institutional Animal Care and Use Committee of Tokyo University of Science (Approval Number: Y16057). All sample collection was performed under isoflurane anesthesia, and all efforts were made to minimize the number of mice used and their suffering.

### Preparation of Serial Brain Sections

Serial sections of mouse brains were obtained according to previously reported methods (Onoda et al., 2020). We randomly chose one male offspring per one dam and collected the brains from the selected offspring at 3 weeks after birth. The offspring mice were put into anesthesia box filled with isoflurane and taken out from the box when they fell asleep. The mice were laid on their back and placed inhalation anesthesia device for isoflurane in their nose. The mice continuously anesthetized by isoflurane were transcardially perfused with 11 ml of phosphate buffered saline (PBS) containing heparin (10,000 units) at a rate of 7 ml/min. After start of the perfusion, right atrium was cut open for drainage. Then, perfusate was switched to 4% paraformaldehyde fixation solution in 0.1 mol/L phosphate buffer at 4°C. Perfusion was performed at a rate of 5 ml/min for 6 min. Volume of the perfusate is 30 ml. The brains were collected after perfusion and post-fixed in 4% paraformaldehyde in 0.1 mol/L phosphate buffer for 24 h.

To prepare frozen sections, the fixed brain tissues were cryoprotected in PBS-sucrose solutions (10% sucrose, 10 h; 20% sucrose, 10 h; 30% sucrose, 30 h) containing 0.1% sodium azide. The brains were then embedded in Tissue-Tek OCT compound (Sakura Finetek Japan Co., Ltd., Tokyo, Japan) and immediately frozen in Histo-Tek Hyfluid (Sakura Finetek Japan Co., Ltd.) at −80°C. Serial sections (5-μm thick or 10-μm thick) were prepared from the frozen blocks using a Tissue-Tek Polar instrument (Sakura Finetek Japan Co., Ltd.) and mounted onto a glass slide for detection of autofluorescence and immunofluorescence. The sections were air-dried for 48 h before staining to prevent moisture interference. Immediately before each staining, the dried frozen sections were submerged in PBS to remove the Tissue-Tek OCT compound.

To prepare paraffin sections, fixed brain tissues were dehydrated using ethanol and xylene. After dehydration, the brains were soaked and embedded in paraffin at 60°C. Serial sections (3-μm thick) were prepared from the paraffin blocks using a sliding microtome (Sakura Finetek Japan Co., Ltd.) and mounted onto a glass slide for Periodic acid-Schiff (PAS) staining, immunohistochemistry, and PAS-immunohistochemical double staining. The serial sections were air-dried for 48 h before staining to adhere firmly to the glass slides. Immediately before each staining, the paraffin sections were deparaffinized using xylene and rehydrated with ethanol and pure water.

### Fluorescence Microscopy for Autofluorescence

A portion of the frozen serial sections was enclosed by cover glass using Prolong Gold Antifade Reagent (P36934, Thermo Fisher Scientific Inc., Weltham, MA, United States). Autofluorescence of PVM granules was evaluated using a fluorescence microscope (Biorevo BZ-9000, Keyence Corporation, Osaka, Japan) with an excitation laser of 488 nm.

### PAS Staining for Brain Tissues

This staining method was different from the common method, because it was modified for brain tissue. PAS staining is mainly used for detection of polysaccharides including glycogen, glycoproteins, and glycolipids. Thus, this staining has been used to evaluate liver diseases associated with glycogen deposition, lung diseases caused by mucin abnormalities, etc. However, the staining is rarely used for histopathological analysis for brain tissues. Therefore, we optimized the staining time of each step and concentration of each reagent to clearly detect and evaluate brain PVMs. A portion of the frozen serial sections and paraffin serial sections were stained using PAS staining to visualize the PVM granules. Rehydrated paraffin sections and frozen sections were oxidized in 1% periodic acid solution for 1 min. After rinsing for 3 min in distilled water, the sections were soaked in cold Schiff reagent for 45 min. The sections were then soaked in sulfurous acid solution three times for 5 min and then rinsed for 3 min in distilled water. Finally, the sections were counterstained with hematoxylin for 1 s, washed in flowing tap water and distilled water, dehydrated in graded alcohol, and cleared in xylene. Coverslips were applied using Permount mounting medium (Thermo Fisher Scientific). These sections were observed using a BX41 microscope (Olympus, Co., Tokyo, Japan) equipped with a digital camera (Olympus).

### Single Immunofluorescence With Autofluorescence Detection

The frozen brain sections were stained using immunofluorescent antibodies following standard methodology to detect the protein expression of macrophage mannose receptor (MMR/CD206) as a selective marker of PVM (Galea et al., 2005). The sections were blocked with 10% normal donkey serum (IHR-8135, Immunobioscience, Mukilteo, WA, United States) in PBS for 1 h at room temperature. The sections were then incubated with primary goat polyclonal anti-MMR antibody (AB_2063012, 1:200 in PBS; code no. AF2535, R&D Systems, Minneapolis, MN, United States) for 20 h at 4°C. After rinsing three times for 5 min per rinse with PBS, sections were incubated with secondary Dylight 649-conjugated donkey anti-goat IgG (AB_1057495, 1:1,000 in PBS; code-no. 605-743-125; Rockland Immunochemicals, Inc. PA, United States) for 180 min at room temperature. The sections were then rinsed three times for 5 min per rinse with PBS and twice for 5 min per rinse with distilled water. Nuclei were counterstained using 4′,6-diamidino-2-phenylindole (DAPI) included in the Prolong Gold Antifade Reagent (P36935, Thermo Fisher Scientific Inc.).

### Double Immunofluorescence

The frozen brain sections were also stained using immunofluorescent antibodies following standard methodology to detect MMR protein expression as a selective marker of PVM (Galea et al., 2005) and platelet endothelial cell adhesion molecule-1 (PECAM-1/CD31) as a selective marker of blood vessels (Nourshargh et al., 2006). The sections were blocked with 10% normal donkey serum (IHR-8135, Immunobioscience) in PBS for 1 h at room temperature. The sections were then incubated with primary goat polyclonal anti-MMR antibody (1:200 in PBS; code no. AF2535, R&D Systems, Minneapolis, MN, United States) for 20 h at 4°C. After rinsing three times for 5 min per rinse with PBS, sections were incubated with secondary Dylight 488-conjugated donkey anti-goat IgG (AB_1057482, 1:1,000 in PBS; code-no. 605-741-125; Rockland Immunochemicals, Inc.). After rinsing three times for 5 min per rinse with PBS, sections were incubated with primary rabbit polyclonal anti-CD31 antibody (AB_726362, 1:100 in PBS; code no. ab28364, Abcam, Cambridge, United Kingdom) for 20 h at 4°C. After rinsing three times for 5 min per rinse with PBS, sections were further incubated with secondary Dylight 649-conjugated donkey anti-rabbit IgG (1:1,000 in PBS; code-no. 611-743-127, Rockland Immunochemicals Inc.) for 180 min at room temperature. The sections were then rinsed three times for 5 min per rinse with PBS and twice for 5 min per rinse with distilled water. Nuclei were counterstained using DAPI included in the Prolong Gold Antifade Reagent (P36935, Thermo Fisher Scientific Inc.).

### PVM Counting Based on MMR Expression

The number of PVMs in the cerebral cortex was manually and blindly quantified based on MMR expression in brain perivascular space using a fluorescence microscope (Biorevo BZ-9000). For each brain obtained from the mice offspring, 20 sections (total 200 μm thickness) were prepared from the longitudinal fissure of the cerebrum along the sagittal plane and subjected to quantitative analysis of MMR-positive PVMs. The cells were confirmed by fluorescence microscopy at ×400-magnification and plotted on ×40-magnified photographs. The MMR-positive PVM number per 1 mm^2^ area was calculated for the cerebral cortex.

### Oil Red O Staining for Brain Tissues

This staining method was different from the common method, because it was modified for brain tissue. Frozen sections were used for Oil Red O staining. At least 10 h prior to staining, 0.5 g of Oil Red O (Sigma Chemical Co., Ltd., WA, United States) was dissolved in 100 ml of 99.5% isopropanol, and the reagent solution was constantly agitated until it was used. The Oil Red O reagent solution was diluted to 60% with pure water, filtered once through filter paper to remove dissolved reagent, and then heated to 37°C. After preparation, the rehydrated frozen sections were soaked in 60% isopropanol for 1 min. After the water in the sections was replaced with isopropanol, the sections were soaked in the prepared Oil Red O reagent solution at 37°C for 30 min. The stained sections were then rinsed twice with 60% isopropanol and distilled water for 3 min. Finally, the sections were counterstained with hematoxylin for 1 s and then washed in flowing tap water and distilled water. After all staining steps, the sections were encapsulated by aqueous mountant (glycerol: PBS = 1 : 1) and cover glass, and the area around the cover glass was sealed with nail polish. These sections were observed using a BX41 microscope (Olympus, Co., Tokyo, Japan) equipped with a digital camera (Olympus).

Oil Red O staining is generally used for the detection of various lipids, especially triglycerides. Because Oil Red O is a non-polar and lipophilic azo dye, it dissolves in intracellular lipids. Therefore, lipid-rich areas are stained darker red because more Oil Red O can dissolve into them.

### Sudan Black B Staining for Brain Tissues

This staining method was different from the common method because it was modified for brain tissue. Frozen sections were used for the Sudan Black B staining. Rehydrated frozen sections were soaked in 70% ethanol for 3 min. After water in the sections was replaced with ethanol, the sections were soaked in Sudan Black B reagent (Muto Pure Chemical) for 20 h. Then, the sections were rinsed with 70% ethanol three times to remove the excess Sudan Black B adsorbed onto the sections. Before the lipid was decolorized, the sections were rinsed in distilled water for 5 min to remove the ethanol. Finally, the sections were encapsulated by an aqueous mountant (glycerol: PBS = 1 : 1) and cover glass, and the area around the cover glass was sealed with nail polish. These sections were observed using a BX41 microscope (Olympus, Co., Tokyo, Japan) equipped with a digital camera (Olympus).

Sudan Black B staining, as well as Oil Red O staining, is one of the most common lipid staining methods. Although Sudan Black B staining does not have a wide staining range compared with Oil Red O staining, it can be used for long-term staining and sensitively detects a small amount of lipids because the dye does not precipitate, unlike Oil Red O. Lipid-rich areas are stained darker blue or black because more Sudan dye can remain in them.

### Masson’s Trichrome Staining for Brain Tissues

This staining method was different from the common method, because it was modified for brain tissue. Paraffin sections were used for Masson’s trichrome staining. Rehydrated paraffin sections were reacted with 10% trichloroacetic acid solution (Muto Pure Chemical Co. LTD., Tokyo, Japan) as a mordant for 10 min and rinsed with flowing tap water for 5 min. The sections were soaked in Caracci hematoxylin reagent (Muto Pure Chemical) for 40 min to stain the nuclei. After staining, a 10-min rinse was performed under flowing tap water to promote color development. The sections were then exposed to Orange G reagent (Muto Pure Chemical) for a very short time (less than 1 s) to stain red blood cells. The sections were immediately washed twice with 1% acetic acid. The sections were soaked in 10-fold diluted Masson’s stain B reagent (Muto Pure Chemical) for 30 s and washed twice with 1% acetic acid water. Then, the sections were soaked in aniline blue reagent (Muto Pure Chemical) for 5 min to stain collagen fibers and washed twice with 1% acetic acid water. Finally, the sections were rinsed with flowing tap water for 5 min and distilled water, dehydrated in graded alcohol, and cleared in xylene. Coverslips were applied using Permount mounting medium (Thermo Fisher Scientific). These sections were observed using a BX41 microscope (Olympus, Co., Tokyo, Japan) equipped with a digital camera (Olympus).

This staining method is mainly used to detect collagen fibers. Nuclei in cells were dyed deep purple. The cytoplasm was dyed red or orange. Collagen fibers and basement membranes were dyed bright blue. Red blood cells were stained light orange.

### PAS-Immunohistochemical Double-Staining for Brain Tissues

Paraffin and frozen sections were used to establish double staining of immunohistochemistry and PAS. In the present study, glial fibrillary acidic protein (GFAP), an astrocyte activation marker (Pekny and Nilsson, 2005; Pekny et al., 2014), was stained with PAS-positive granules of PVMs in the same brain section. First, endogenous peroxidase in the brain sections was deactivated with 0.3% hydrogen peroxide (H_2_O_2_) in PBS for 30 min at room temperature. After blocking with 10% normal donkey serum (IHR-8135, Immunobioscience) in PBS for 1 h at room temperature, the sections were incubated in primary rabbit polyclonal anti-GFAP antibody (AB_10013382, 1:1,000 in PBS-0.01%Triton X, code-no. Z0334, DakoCytomation Co.) for 16 h at 4°C. After rinsing three times for 5 min per rinse with PBS-0.01%Triton X, sections were further incubated in secondary biotinylated donkey anti-rabbit IgG (1:1,000 in PBS-0.01%Triton X Chemicon, Temecula, CA, United States; 1:1,000) for 120 min at room temperature and rinsed three times for 5 min per rinse with PBS-0.01%Triton X.

After antibody incubation, the sections were treated with 1% periodic acid solution for 3 min, rinsed with distilled water for 1 min, and soaked in cold Schiff reagent for 60 min. Sections were submerged in sulfurous acid solution three times for 3 min each, and then rinsed with distilled water for 1 min.

After PAS staining step, sections were treated with an avidin-biotin-peroxidase complex (1:400 in pure water, Vectastain ABC peroxidase kit, Vector Laboratories Inc., CA, United States) for 120 min and reacted in a solution of 0.02% 3,3′-diaminobenzidene (DAB) or DAB-cobalt substrate in 0.1 mol/L Tris–HCl buffer (pH 7.6). After the DAB reaction, the sections were treated with 0.01% H_2_O_2_ for 20 min to detect peroxidase activity. When DAB substrate was used, immunoreactivity was visible as light brown staining. In contrast, immunoreactivity was visible as dark brown (black) staining if the DAB-cobalt substrate was chosen. Sections were then washed in PBS, dehydrated in graded alcohol, cleared in xylene, and mounted on coverslips with permount mounting medium (Thermo Fisher Scientific Inc.). These sections were observed using a BX41 microscope (Olympus) equipped with a digital camera (Olympus).

### Immunohistochemistry

Immunohistochemistry was performed according to the double staining method described above, without PAS staining step. This was performed to compare the ease of detection of brain perivascular injuries with PAS-immunohistochemical double-staining. Endogenous peroxidase in the paraffin sections was deactivated with 0.3% H_2_O_2_ in PBS for 30 min at room temperature. After blocking with 10% normal donkey serum (IHR-8135, Immunobioscience) in PBS for 1 h at room temperature, the sections were incubated in primary rabbit polyclonal anti-GFAP antibody (1:1,000 in PBS-0.01%Triton X, code-no. Z0334, DakoCytomation Co.) for 16 h at 4°C. After rinsing three times for 5 min per rinse with PBS-0.01%Triton X, sections were further incubated with secondary biotinylated donkey anti-rabbit IgG (AB_92587, 1:1,000 in PBS-0.01%Triton X Chemicon, Temecula, CA, United States; 1:1,000) for 120 min at room temperature and rinsed three times for 5 min per rinse with PBS-0.01%Triton X. The sections were treated with an avidin-biotin-peroxidase complex (1:400 in pure water, Vectastain ABC peroxidase kit, Vector Laboratories Inc., CA, United States) for 120 min and reacted in a solution of 0.02% DAB substrate in 0.1 mol/L Tris–HCl buffer (pH 7.6). After the DAB reaction, the sections were treated with 0.01% H_2_O_2_ for 20 min to detect peroxidase activity. Immunoreactivity was visible as light brown staining. Sections were then washed in PBS, dehydrated in graded alcohol, cleared in xylene, and mounted on coverslips with permount mounting medium (Thermo Fisher Scientific Inc.). These sections were observed using a BX41 microscope (Olympus) equipped with a digital camera (Olympus).

### Quantification of Monoamine and its Metabolites in Each Brain Region

The amounts of each neurotransmitter and its metabolite in various brain regions were evaluated according to previously reported methods (Okada et al., 2013). As neurotransmitters, monoamines and their metabolites were detected and quantified using high-performance liquid chromatography. Brains were collected from 3-week-old pups (n = 8/group). Male mice were used in the present study because neurotransmitter levels are altered at different times during the estrous cycle in female mice (Xiao and Becker, 1994; Dazzi et al., 2007; Orthofer et al., 2020). Serial coronal sections (2 mm thick) were acquired using a rodent brain slicer (Muromachi Kikai, Tokyo, Japan) and nine brain regions were obtained: the prefrontal cortex (PFC), nucleus accumbens (NAcc), paraventricular hypothalamic nucleus (PVN), ventral tegmental area (VTA), dorsal raphe nucleus (DRN), caudate-putamen/neostriatum (Cpu), central nucleus of amygdala (CEA), and medullary reticular formation (MRF). These separated tissues were immediately frozen in liquid nitrogen and stored at −80°C until use.

Each brain tissue was homogenized in cold perchloric acid containing 0.1 mol/L ethylenediaminetetraacetic acid (2Na). Isoproterenol (100 ng) was added to the lysate as an internal standard. The lysate was centrifuged at 20,000 × g for 15 min at 4°C to remove insoluble debris, and the supernatants were collected for analysis. The pH of the supernatant was adjusted to 3.0, using 1 mol/L sodium acetate, and stored at −80°C until use. The pH-adjusted supernatant (10 μL) was injected into an high-performance liquid chromatography system with electrochemical detection (Eicom Co., Kyoto, Japan). Monoamines and their metabolites were separated using a C18 reverse-phase column (Eicompak SC-5ODS, Eicom) with a mobile phase containing sodium acetate and citric acid. The mobile phase was prepared as follows: 0.1 mol/L sodium acetate was mixed with 0.1 mol/L citric acid in a 10:9 ratio, and was adjusted to pH 3.5 (0.1 mol/L sodium acetatecitric acid buffer), mixed with methanol in a ratio of 85:15 and then supplemented with sodium 1-octanesulfonate (100 mg/L), ethylenediaminetetraacetic acid (2Na) (5 mg/ml). As monoamines, dopamine (DA), noradrenaline (norepinephrine, NA), adrenaline (epinephrine, Ad), and serotonin (5-Hydroxytryptamine, 5-HT) were measured. We also quantified 3,4-dihydroxyphenylacetic acid (DOPAC), 3-methoxytyramine (3-MT), and homovanillic acid (HVA) as metabolites of DA, normetanephrin (NM) as a metabolite of NA, 3-Methoxy-4-hydroxyphenylglycol (MHPG) as a metabolite of NA and Ad, and 5-hydroxyindole-3-acetic acid (5-HIAA) as a metabolite of serotonin.

### Behavioral Tests

The open field test and elevated plus maze test were performed to evaluate brain function. We randomly chose one male offspring per one dam and collected the brain from the selected offspring at 8 weeks after birth. This age was selected because mice should normally be 8–24 weeks old at the time of testing to evaluate the maturation and development of brain function (Crawley, 2008), except when examining the specialized tests for newborn mice. The chosen mice were transferred to a soundproof room (100 lx) for behavioral test an hour before testing of the first animal to habituate them to the condition of the room. The control and CB-NP groups were tested alternately.

To examine spontaneous locomotor activity of the mice, we counted the number of center square entries and reared them in the open field (100 cm square), a novel environment for mice, after a mouse was placed in its center. Based on guideline of the National Toxicology Program, behavior was measured every 5 min for a total of 30 min. The behavior (total distance, number of entries into center square, and number of rearing) of the mice was recorded and analyzed using a digital counter with Video Tracking Interface software, version 1.4 (Home Cage Video Tracking System, MED Associates Inc., VT, United States).

Anxiety-like behavior was evaluated by elevated plus maze test. The number of entries into open arms and time in the open arms in 5-min were measured. The apparatus consisted of two open arms (25 × 5 × 0.5 cm), two closed arms (25 × 5 × 16 cm), and a central area (5 × 5 × 0.5 cm). The distance between each arm and the floor was 50 cm. A mouse was placed in the central area, with the head facing the closed arms. The behavior was recorded and analyzed using a digital counter with video tracking interface software, version 1.4 (Home Cage Video Tracking System).

After each trial of behavioral tests, the entire apparatus was cleaned with disinfectant ethanol and weakly acidic hypochlorite water to remove the odor of prior mice and prevent any olfactory-based bias.

### Statistical Analysis

All data are presented as mean ± standard deviation, and the levels of significance are cited. R version 4.1.2 (https://www.r-project.org/) was used for statistical analyses. The number and sex ratio of newborns per dam, body weight of offspring, amounts of neurotransmitters, and results of behavioral tests were analyzed using Welch’s *t*-test. The level of significance was set at *p* < 0.05.

## Results and Discussion

### Identification of PVMs and its Properties

**FIGURE 1:**
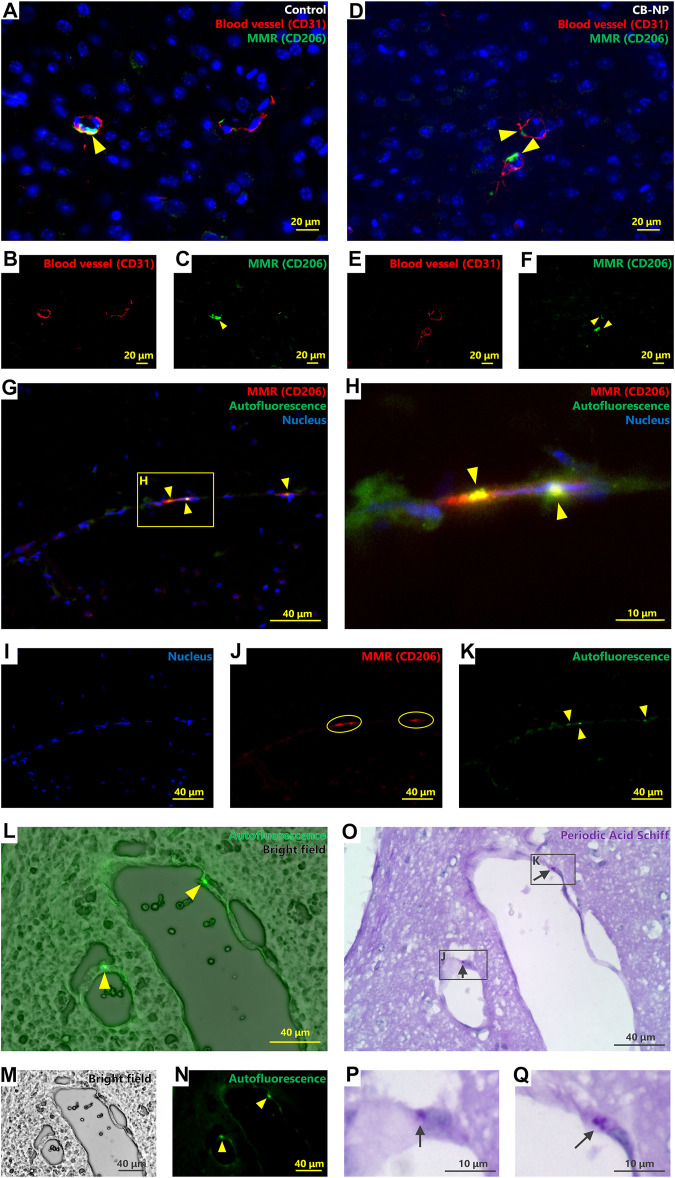
Identification and characterization of brain perivascular macrophages (PVMs). Autofluorescence, macrophage mannose receptor (MMR) expression, and Periodic acid-Schiff (PAS) stainability were evaluated to detect and identify PVMs using brain sections from the control group. **(A–F)** Immunofluorescence of macrophage mannose receptor (MMR) and CD31, a blood vessel marker. **(A,D)** are merged pictures of B and C, and E and F, respectively. Arrowheads indicate MMR-positive cells. MMR-positive cells were observed only around the blood vessels in the cerebral cortex. Activated microglia (MMR-positive microglia) were not detected in either control **(A–C)** or CB-NP groups **(D–F) (G–K)** Immunofluorescence of MMR and autofluorescence. H shows an enlarged view of **(G)**. **(G)** is a merged picture of **(I–K)**. Arrowheads indicate autofluorescence granules. Circles indicate cells expressing the MMR. Autofluorescence was detected in MMR-positive cells. **(L-Q)** Localization of autofluorescence granules and PAS-positive granules. **(L)** is a merged picture of **(M,N)**. Arrowheads indicate autofluorescence of PVM granules. Brain section in **(O)** was serial section of **(L–N)**. **(P,Q)** show enlarged views of a part of **(O)**. Arrows indicate PAS-positive granules. The localization of autofluorescence and PAS-positive granules was consistent.

First, we confirmed the properties of PVMs to be able to correctly detect them. Previous studies have already revealed that MMR (CD206) is a specific marker of PVMs in brain (Galea et al., 2005; Zhang et al., 2017). In fact, MMR-positive cells were detected only around blood vessels in the brains of the control group (Figures 1A–F).

There is cell called Mato cells (fluorescent granular perithelial cell) in some literatures, as a type of cells which possess lysosomal granules stained with PAS staining (Mato et al., 2002; 2009) and emitting strong autofluorescence with a 488-nm excitation laser (Mato et al., 1980; Ookawara et al., 1996) in the perivascular region. Based on their properties and morphologies, Mato cells are considered to be identical to PVMs (Williams et al., 2001; Yang et al., 2019); however, the precise identity of PVMs and Mato cells has not been defined (Faraco et al., 2017). Thus, we evaluated the localization of MMR expression, PAS-stained granules, and autofluorescent granules around blood vessels using serial sections (PAS-stained and non-stained sections) to confirm that PVM and Mato cells are the same cells. Autofluorescence was observed in the MMR-positive cells (Figures 1G–K). Furthermore, localization of autofluorescent granules and PAS-stained granules was completely consistent (Figures 1L–Q). This is the first study to show that the PAS-stained granules around brain blood vessels are identical to lysosomal granules exhibiting autofluorescence included the MMR-positive cells. Therefore, the results suggest that PVMs and Mato cells are the same, and these cells can be detected by PAS staining.

### Quantification of MMR-Positive PVMs and Evaluation of MMR-Positive Microglia

While PVMs constantly express MMR with or without abnormalities such as inflammation and injuries, activated microglia observed in abnormal brains express MMR (Zhang et al., 2017). Thus, the MMR-positive cells in the brains of offspring maternally exposed to CB-NP were investigated in comparison to those of the control group. The difference in their localization can be used to determine the cell. Microglia are found in the brain parenchyma, whereas PVM are found in the only perivascular space surrounded by glial limiting membranes and vascular endothelial cells. Only MMR-positive cells in the perivascular space can be determined to be PVM.

**FIGURE 2:**
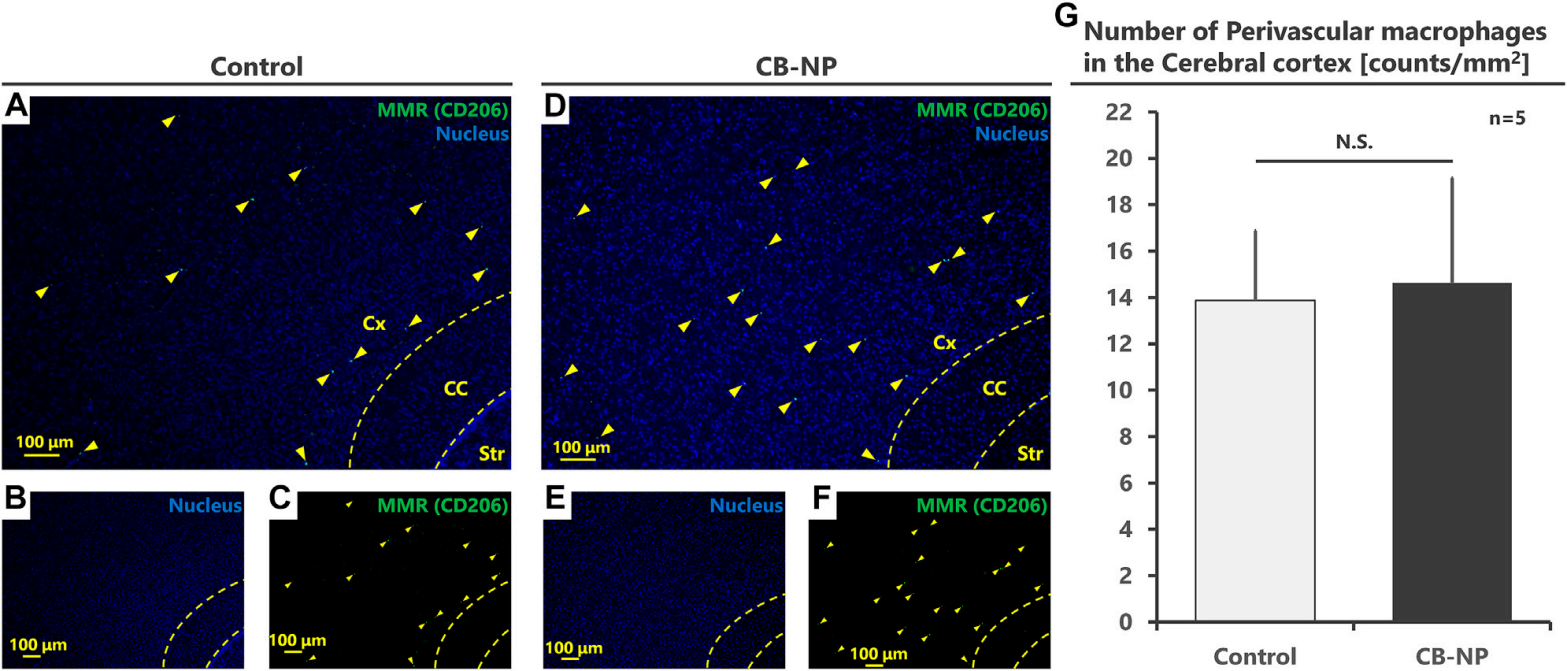
Quantification of the number of perivascular macrophages (PVMs). The number of PVMs in the cerebral cortex was counted and compared between the control and CB-NP exposure groups. **(A–F)** Low-magnification images to quantify the number of PVMs using MMR expression. **(A,D)** are merged pictures of **(B,C,E,F)**, respectively. Arrowheads indicate MMR-positive PVMs. **(G)** Number of PVMs in the cerebral cortex. Values are expressed as the mean ± SD. Abbreviation: Carbon black nanoparticle (CB-NP), Cerebral cortex (Cx), Corpus callosum (CC), and Striatum (Str), Standard deviation (SD).

MMR-positive PVMs were detected in the brain perivascular area, but activated microglia (MMR-positive microglia) were not detected in the brain parenchyma of either the control (Figures 1A–C) or CB-NP groups (Figures 1D–F). Since there were no MMR-positive microglia, quantification of PVMs in the cerebral cortex was performed based on MMR expression. No significant differences in the number of MMR-positive PVMs were observed between the control and CB-NP groups (Figure 2). The results suggested that maternal exposure to low-dose CB-NP used in the present study did not affect microglial activation or the number of PVMs in the brain.

### Denaturation of PVMs and its Easy Detection

**FIGURE 3:**
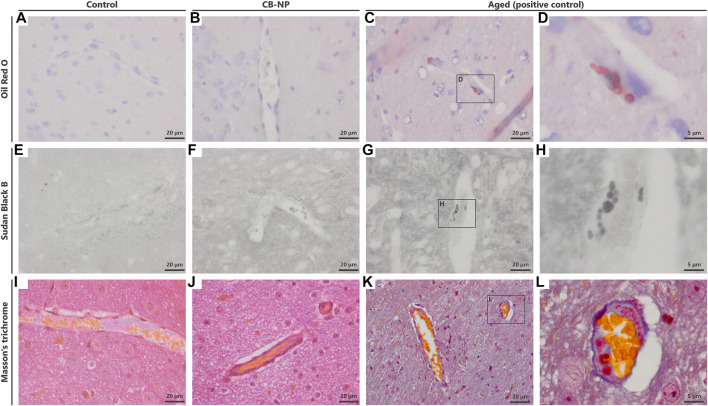
Detection of histopathological changes in perivascular macrophages (PVMs). To detect the denaturation of PVMs, Oil Red O staining, Sudan Black B staining, and Masson’s trichrome staining were performed. **(A–D)** Light micrographs of brain sections stained with Oil Red O. Blue and red indicate the nucleus and accumulation of lipid, respectively. **(E–H)** Light micrographs of brain sections stained with Sudan Black B. **(I–L)** Light micrographs of brain sections stained with Masson’s trichrome stain. **(D,H,L)** are enlarged pictures of **(C,G,K)**, respectively. These staining could stain PVMs with severe denaturation in the brains of aged mice but did not detect mild denaturation of PVMs in the brains of both the control and carbon black nanoparticle exposure groups.

**FIGURE 4:**
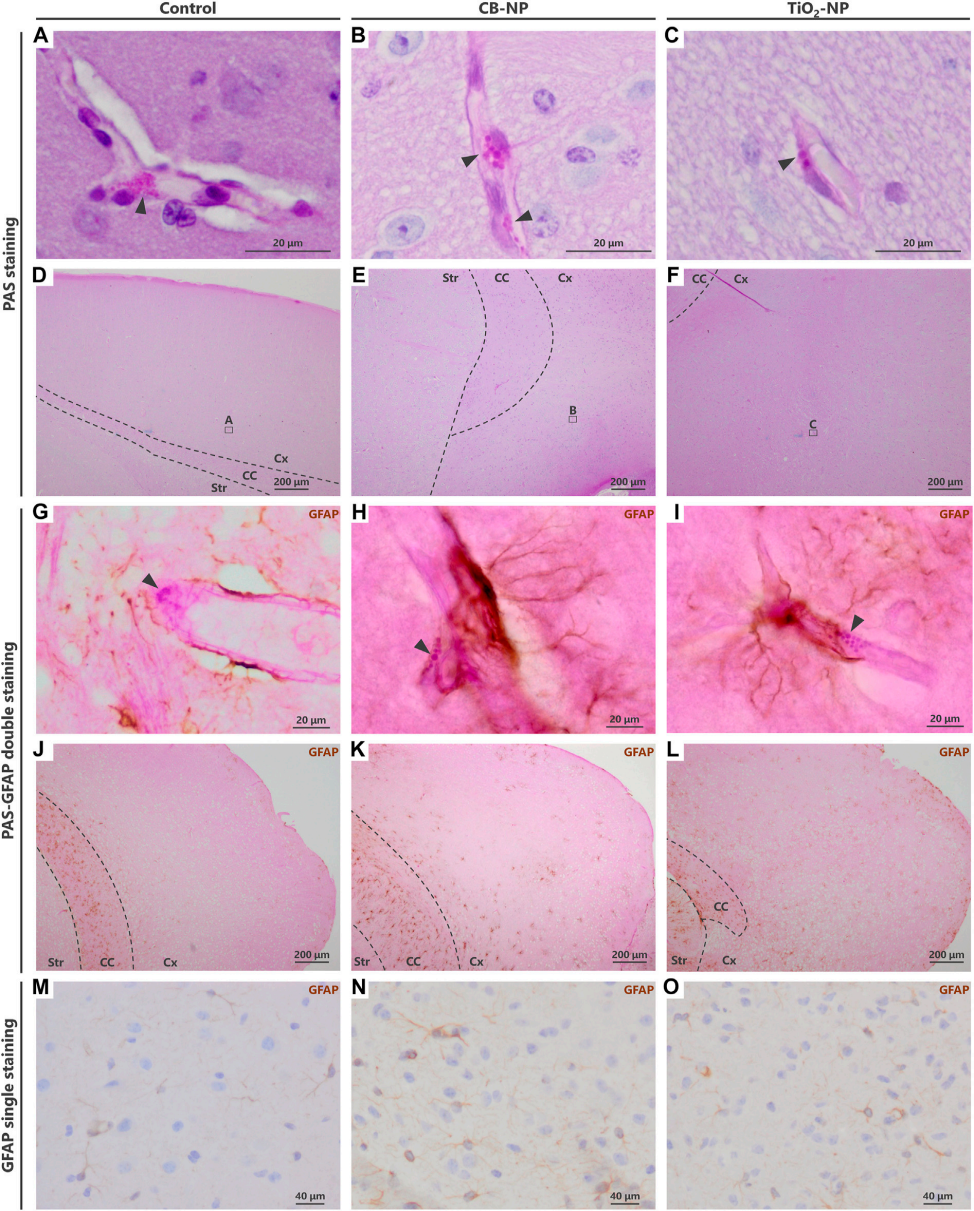
Visualization of enlargement of granules in denatured perivascular macrophages (PVMs). To detect the denaturation of PVMs, their granules were visualized by Periodic acid-Schiff (PAS) staining. In contrast to PAS single staining, PVM denaturation was easily detected by PAS-immunohistochemical double staining. **(A–F)** Light micrographs of the PVMs stained with PAS staining. **(A–C)** are enlarged pictures of **(D–F)**, respectively. Arrowheads indicate PAS-positive granules in PVMs. Enlargement of the granules was observed after maternal exposure to the nanoparticles. In PAS single staining, the entire brain tissue is stained pink and PVM granules (approximately 1–3 μm) are inconspicuous, making it difficult to detect and evaluate the granules. **(G–L)** Light micrographs of PVMs and astrocytes stained with PAS-immunohistochemical double staining. **(G–I)** are enlarged pictures of **(J,K)**, respectively. Arrowheads indicate PAS-positive granules in PVM, and brown indicates high expression of glial fibrillary acidic protein (GFAP) stained with immunohistochemistry. In the cerebral cortex, this protein, an astrocyte activation marker, is highly expressed in areas where histopathological changes occur. By focusing on the location of the browns stained with double staining, brain perivascular injuries, including PVM denaturation, can be found more easily than PAS single staining. **(M–O)** Immunohistochemistry of GFAP. It is difficult to detect perivascular injuries using immunohistochemistry alone.

Previous studies have suggested that PVM granules are sensitively denatured by weak stimuli (Onoda et al., 2014; 2017a). To evaluate the denaturation of PVMs and their granules, we visualized them using PAS staining, Oil Red O staining, Sudan Black B staining, and Masson’s trichrome staining methods in accordance with previous studies (Mato et al., 1983: Mato and Ookawara, 1979). However, Oil Red O, Sudan Black B staining, and Masson’s trichrome staining were able to stain only PVMs with severe denaturation, such as lipid accumulation, which was observed in aged mice (Figures 3C,D,G,H,K,L). In other words, these staining methods could not be used to evaluate the histopathological differences in PVMs between the control and low-dose CB-NP groups (Figures 3A,B,E,F,I,J). In contrast to these staining, PAS staining captured enlargement of PVM granules, their typical mild denaturation (Mato et al., 2002), which occurred due to maternal exposure to low-dose CB-NP (Figures 4A,B). In contrast, when the cell receives severe damages, loss of lysosomal granules or cell death will be induced (Mato et al., 2002). In addition, the same PVM denaturation was detected in the brains of offspring maternally exposed to low-dose TiO_2_-NP (Figure 4C). As the denaturation of PVMs progresses, cell death is eventually induced and their number decreases (Mato et al., 2002; Onoda et al., 2014). Therefore, these results indicate that PAS staining can detect mild histopathological abnormalities of PVMs before their cell number changes, as previously shown (Figure 2). Enlargement of PVM granules may be a sensitive endpoint for the assessment of neurotoxic effects.

Although the evaluation of PVM granules using PAS staining may be an effective approach for the sensitive detection of brain injuries, it is difficult for novice pathologists without sufficient experience to detect and determine their slight histopathological changes. Since PAS staining turned the entire brain tissue pink, its small granules were inconspicuous (Figures 4D–F). To solve this problem, we combined PAS staining with immunohistochemistry.

Previous study has revealed that the expression of GFAP, an astrocyte activation marker, was increased around PVMs with enlarged granules (Onoda et al., 2014). In fact, PVMs and surrounding astrocytes are closely interrelated with each other (Morita-Takemura et al., 2019). Since blood vessels with high expression of astrocytic GFAP in their surroundings are very likely to have denatured PVMs, visualizing the GFAP expression around blood vessels leads to an easy determination of the vessels to be observed. The PAS-immunohistochemical double-staining method has allowed the assessment of the morphology of PVMs and their granules, expression of target molecules including GFAP, and their relationship in the same brain section. Using this double staining for PAS and GFAP in the present study, we were able to easily and quickly find enlargement of PVM granules in the blood vessels with high expression of GFAP in the CB-NP and TiO_2_-NP exposure groups compared to the control group (Figures 4G–L), which is consistent with previous findings (Onoda et al., 2014). These findings suggest that this double staining will help novices in histopathological analysis to easily identify mild injuries in the brain, including developmental neurotoxic effects. In contrast, using immunohistochemistry for GFAP alone, it was difficult to identify the brain blood vessels and perform histological observations focused on them (Figures 4M–O).

### Verification of Various Patterns of PAS-Immunohistochemical Double Staining

Not only GFAP, the PAS-immunohistochemical double-staining can be used to visualize other molecules using appropriate antibodies. The double staining method will contribute to the investigation of the properties of PVMs and the relationship between the PVMs and the surrounding cells. Therefore, we examined various staining procedures for double staining to determine the best staining condition. As a result of the test, the best staining was obtained by adding the PAS staining step between the secondary antibody incubation and the avidin/biotinylated enzyme complex reaction (Table 1). There are several important points in this staining method that appeared in this test.

**FIGURE 5:**
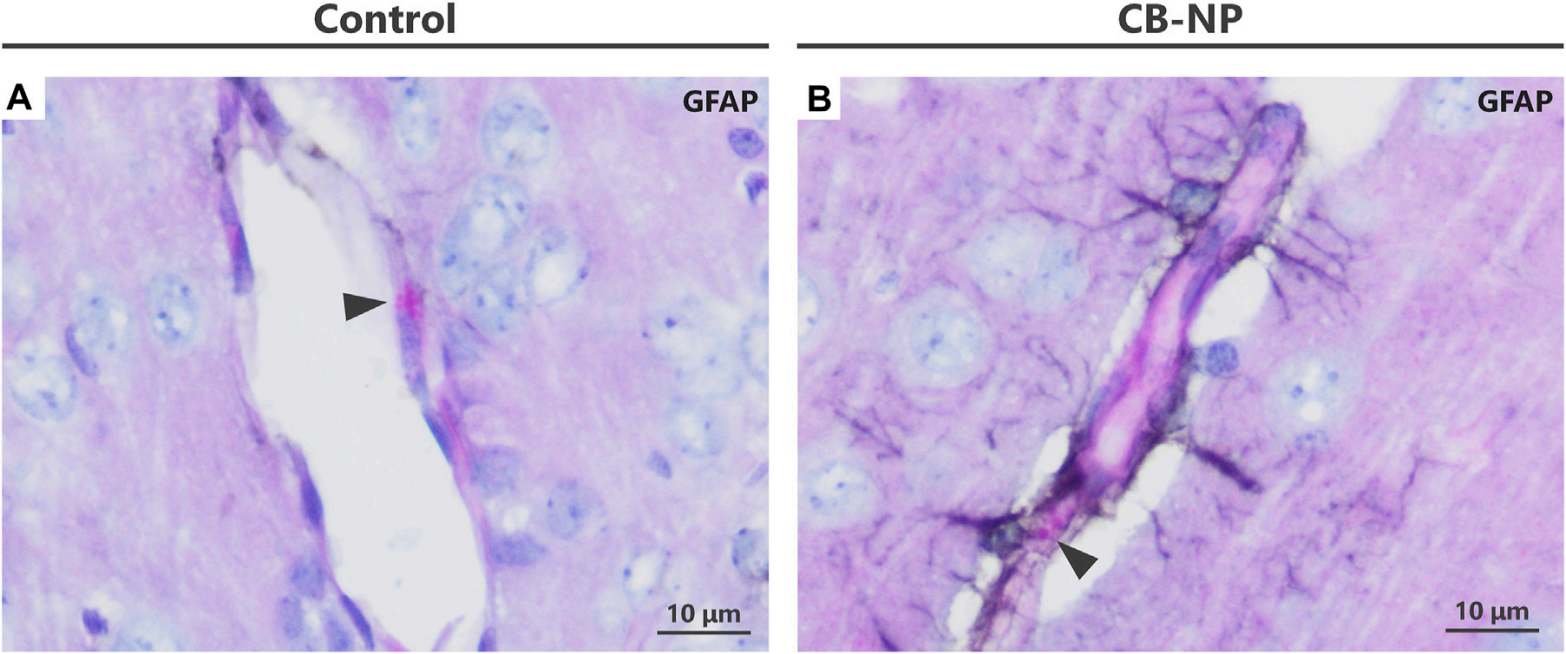
PAS-immunohistochemistry using diaminobenzidine (DAB) with cobalt. When the DAB substrate was mixed with cobalt, the brown turned black or dark brown. **(A,B)** Light micrographs of PVMs and astrocytes stained with PAS-immunohistochemical double staining using a DAB-cobalt substrate. The astrocyte end-feet were stained black. This staining with DAB-cobalt is suitable when the staining of astrocytes needs to be emphasized. Arrowheads indicate PAS-positive granules in PVMs.

1) This double staining could be used on both paraffin and frozen sections.
2) No decrease in PAS staining performance due to antigen retrieval was observed. When antigen retrieval is needed, it should be performed before the deactivation of endogenous peroxidase.
3) Depending on the target molecule, avidin and biotin blocking may be required.
4) Rinsing with PBS containing surfactants such as TritonX slightly degraded PAS staining performance. After PAS staining step, the sections were rinsed with PBS that did not contain surfactants or contained very little surfactant.
5) H_2_O_2_ slightly faded the color of PAS. Care must be taken when reacting highly concentrated H_2_O_2_ for a long time.
6) Nuclear staining is not recommended because it makes observation of PVMs and their surroundings difficult.
7) The color of the target molecules turned black when the DAB substrate contained cobalt (Figures 5A,B). The DAB-cobalt substrate can be used when there is a need to observe the expression of the target molecule more clearly.

**TABLE 1:** Examination of various methods of Periodic acid-Schiff and immunohistochemical double-staining and its staining results.

Staining step	Normal PAS staining for brain tissues	Normal immunohistochemistry	Evaluation of PAS-immunohistochemistry double staining					
			1	2	3	4	5	6
1	Deparaffinization or de-O.C.T. Compound	Deparaffinization or de-O.C.T. Compound	Deparaffinization or de-O.C.T. Compound	Deparaffinization or de-O.C.T. Compound	Deparaffinization or de-O.C.T. Compound	Deparaffinization or de-O.C.T. Compound	Deparaffinization or de-O.C.T. Compound	Deparaffinization or de-O.C.T. Compound
2	Rehydration	Rehydration	Rehydration	Rehydration	Rehydration	Rehydration	Rehydration	Hydration
3	Rinse	Rinse	Rinse	Rinse	Rinse	Rinse	Rinse	Rinse
4	Periodic acid oxidation	Antigen retrieval (optional)	Antigen retrieval (optional)	Antigen retrieval (optional)	Antigen retrieval (optional)	Antigen retrieval (optional)	Antigen retrieval (optional)	antigen retrieval (optional)
5	Rinse	Rinse	Rinse	Rinse	Rinse	Rinse	Rinse	Rinse
6	Cold Schiff staining	Deactivation of endogenous peroxidase	Periodic acid oxidation	Deactivation of endogenous peroxidase	Deactivation of endogenous peroxidase	Deactivation of endogenous peroxidase	Deactivation of endogenous peroxidase	Deactivation of endogenic peroxidase
7	Sulfurous acid solution	Rinse	Rinse	Rinse	Rinse	Rinse	Rinse	Rinse
8	Rinse	Pre-blocking	Cold Schiff staining	Periodic acid oxidation	Pre-blocking	Pre-blocking	Pre-blocking	Pre-blocking
9	Counterstaining (optional)	Primary antibody	Sulfurous acid solution	Rinse	Primary antibody	Primary antibody	Primary antibody	Primary antibody
10	Rinse	Rinse	Rinse	Cold Schiff staining	Rinse	Rinse	Rinse	Rinse
11	Dehydration and clear	Biotinylated	Deactivation of endogenous peroxidase	Sulfurous acid solution	Periodic acid oxidation	Biotinylated	Biotinylated	Biotinylated
		Secondary antibody				Secondary antibody	secondary antibody	secondary antibody
12	Mounting and coverslip	Rinse	Rinse	Rinse	Rinse	Rinse	Rinse	Rinse
13		Avidin/biotinylated enzyme complex	Pre-blocking	Pre-blocking	Cold Schiff staining	Periodic acid oxidation	Avidin/biotinylated enzyme complex	Avidin/biotinylated enzyme complex
14		Rinse	Primary antibody	Primary antibody	Sulfurous acid solution	Rinse	Rinse	Rinse
15		DAB substrate and hydrogen peroxide	Rinse	Rinse	Rinse	Cold Schiff staining	DAB substrate and hydrogen peroxide	Periodic acid oxidation
16		Rinse	Biotinylated	Biotinylated	Biotinylated	Sulfurous acid solution	Rinse	Rinse
			Secondary antibody	Secondary antibody	Secondary antibody			
17		Counterstaining (optional)	Rinse	Rinse	Rinse	Rinse	Periodic acid oxidation	DAB substrate and hydrogen peroxide
18		Rinse	Avidin/biotinylated enzyme complex	Avidin/biotinylated enzyme complex	Avidin/biotinylated enzyme complex	Avidin/biotinylated enzyme complex	Rinse	Rinse
19		Dehydration and clear	Rinse	Rinse	Rinse	Rinse	Cold Schiff staining	Cold Schiff staining
20		Mounting and coverslip	DAB substrate and hydrogen peroxide	DAB substrate and hydrogen peroxide	DAB substrate and hydrogen peroxide	DAB substrate and hydrogen peroxide	Sulfurous acid solution	Sulfurous acid solution
21			Rinse	Rinse	Rinse	Rinse	Rinse	Rinse
22			Counterstaining (optional)	Counterstaining (optional)	Counterstaining (optional)	Counterstaining (optional)	Counterstaining (optional)	Counterstaining (optional)
23			Rinse	Rinse	Rinse	Rinse	Rinse	Rinse
24			Dehydration and clear	Dehydration and clear	Dehydration and clear	Dehydration and clear	Dehydration and clear	Dehydration and clear
25			Mounting and coverslip	Mounting and coverslip	Mounting and coverslip	Mounting and coverslip	Mounting and coverslip	Mounting and coverslip
Immunohistochemistry	**—**	**+++**	**++**	**++**	**—**	**+++**	**—**	**—**
PAS staining	**+++**	**—**	**—**	**+**	**+**	**+++**	**+**	**—**

### Sensitivity of PVM Denaturation Compared to Other End-points

In the present study, we have emphasized that PVMs and their surrounding astrocytes sensitively respond to even slight stimuli, and their histological changes may be a potential end-point to evaluate toxic effects on the developing brain. In fact, maternal exposure to low-dose CB-NP induced the denaturation of PVMs with astrocyte activation in the brain of offspring; however, microglial activation was not induced by the exposure. In addition to the results, we evaluated the effects of exposure to low-dose CB-NP on the phenotypes of offspring mice.

**FIGURE 6:**
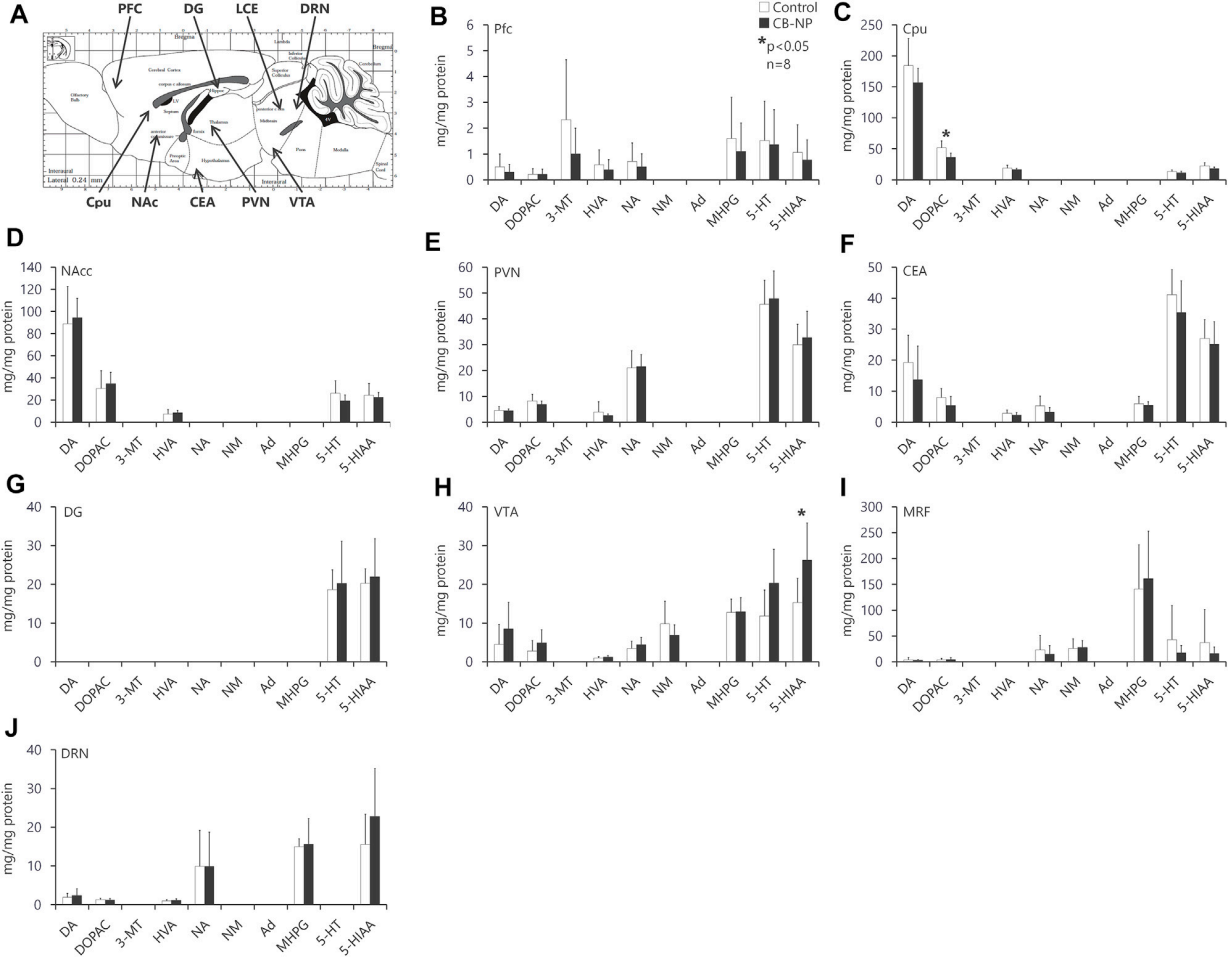
Levels of each neurotransmitter and their metabolite in each brain region. The effect of maternal carbon black nanoparticle exposure on each neurotransmitter and its metabolite in each brain region was evaluated. **(A)** Evaluated brain regions in mice offspring. The image indicates the sagittal section. **(B–J)** Amount of dopamine (DA), noradrenaline (norepinephrine, NA), adrenaline (epinephrine, Ad), serotonin (5-Hydroxytryptamine, 5-HT), 3,4-dihydroxyphenylacetic acid (DOPAC), 3-methoxytyramine (3-MT), homovanillic acid (HVA), normetanephrin (NM), 3-Methoxy-4-hydroxyphenylglycol (MHPG), and 5-hydroxyindole-3-acetic acid (5-HIAA) in the prefrontal cortex (PFC), nucleus accumbens (NAcc), paraventricular hypothalamic nucleus (PVN), ventral tegmental area (VTA), dorsal raphe nucleus (DRN), caudate-putamen/neostriatum (Cpu), central nucleus of amygdala (CEA), and medullary reticular formation (MRF), respectively. Values are expressed as the mean ± standard deviation. *n* = 8. The white column indicates the control group and the black column indicates the carbon black nanoparticle exposure group.

**FIGURE 7:**
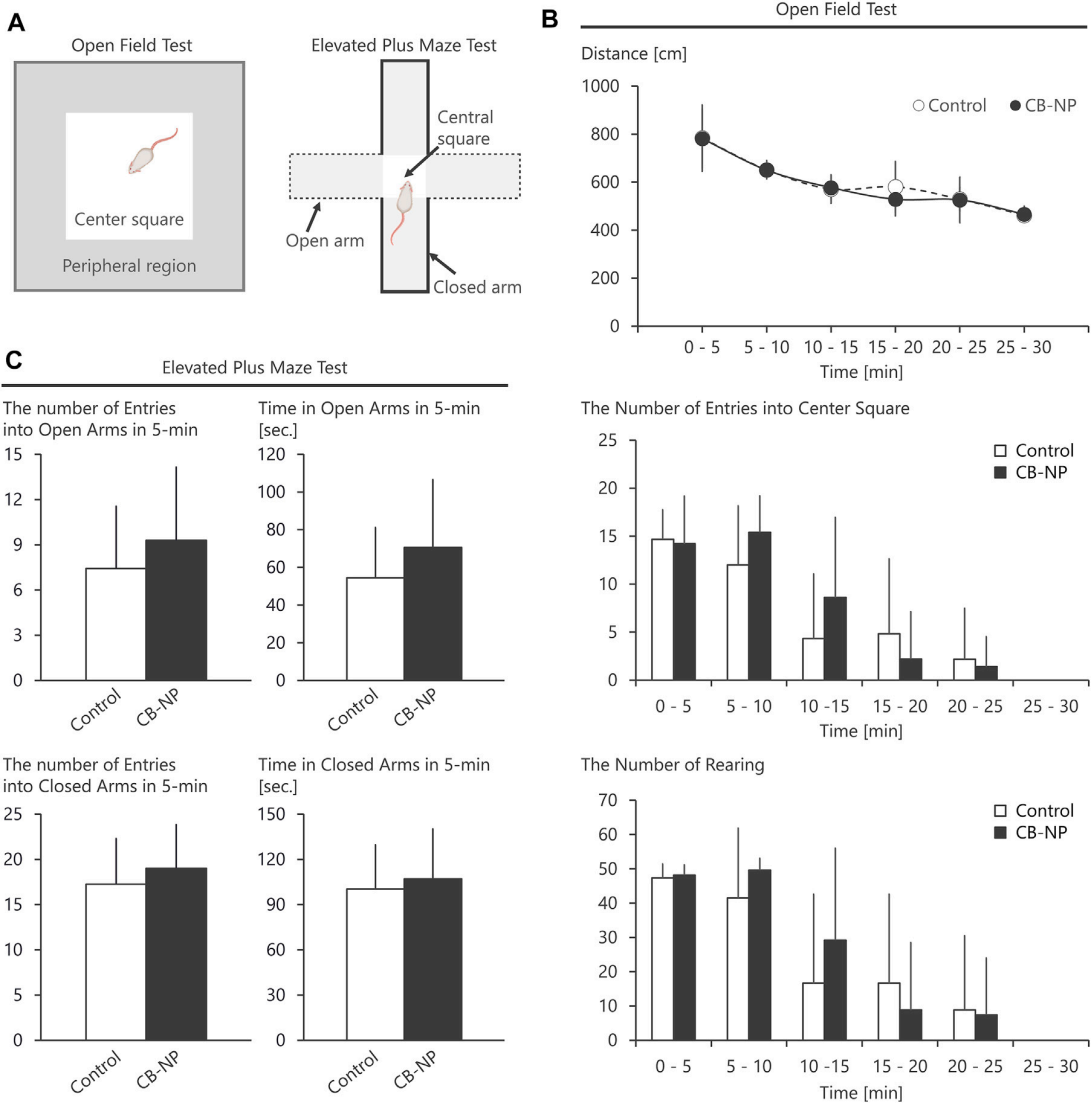
Behavioral tests using open field and elevated plus maze. The effect of maternal carbon black nanoparticle exposure on behavior associated with spontaneous locomotor activity and anxiety-like behavior was evaluated. **(A)** Schematic diagrams of the open field test and the elevated plus maze test. **(B)** Total distance, the number of visits to the center square of the open field, and the number of rearing in the open field. **(C)** The number of visits to open arms or closed arms in the elevated plus maze during 5-min and spending time [s] in the open arms or closed arms for 5-min. Values are expressed as the mean ± standard deviation. *n* = 8. The white column indicates the control group and the black column indicates the carbon black nanoparticle exposure group.

First, there was no significant difference among each group in the number and sex ratio of offspring at birth (Table 2) or body weight at postnatal days 1, 7, and 21 (Table 3). Next, the amounts of four types of neurotransmitters and six types of metabolites in the nine brain regions of the control and CB-NP groups were measured and compared (Figure 6A). Although there were significant differences only in DOPAC, a metabolite of DA, in Cpu and 5-HIAA, a metabolite of 5-HT, and in VTA (Figures 6C,H), there were no significant differences in neurotransmitters and their metabolites in other brain regions (Figures 6A–J). In particular, significant differences in four types of monoamines, DA, NA, Ad, and 5-HT, were not observed in any brain region. This result suggests that the effect of low-doses of CB-NP exposure on neural functions related to neurotransmission may be minimal. Lastly, we evaluated the effects of low-dose CB-NP exposure on behavioral functions associated with spontaneous locomotor activity and anxiety-like behavior (Figure 7A). In the open field test to assess spontaneous locomotor activity in the novel environment, there were no significant differences between the control and CB-NP groups in the total distance, number of visits to the center square, and the number of rearing (Figure 7B). In addition, the elevated plus maze test for the evaluation of anxiety-like behavior showed no significant differences between the control and CB-NP groups in the number of visits and spending time in open or closed arms (Figure 7C). Abnormal behavioral function caused by CB-NP exposure was not observed, in the same was for the neurotransmitter levels.

**TABLE 2:** Number and sex ratio of offspring.

Group	Number of dams	Number of offspring per mother mouse	Sex ratio (%) [male/(male + female) x 100]
Control	13	14.39 ± 1.92	52.19 ± 3.09
CB-NP	13	13.62 ± 2.13	51.88 ± 2.38
TiO_2_-NP	13	13.81 ± 1.78	51.63 ± 2.81

Abbreviation: Carbon black nanoparticle (CB-NP), Titanium dioxide nanoparticle (TiO_2_-NP).

**TABLE 3:** Body weight (g) of offspring at postnatal days (PND) 1, 7, and 21.

Group	PND 1		PND 7		PND 21	
	Male	Female	Male	Female	Male	Female
Control	1.79 ± 0.23	1.71 ± 0.15	4.81 ± 0.82	4.93 ± 0.63	12.91 ± 1.31	13.91 ± 1.52
CB-NP	1.88 ± 0.16	1.76 ± 0.19	4.94 ± 0.61	4.91 ± 0.94	13.89 ± 0.91	14.08 ± 1.73
TiO_2_-NP	1.81 ± 0.19	1.87 ± 0.21	4.89 ± 0.79	5.06 ± 0.88	13.18 ± 1.17	14.41 ± 0.85

Abbreviation: Carbon black nanoparticle (CB-NP), Titanium dioxide nanoparticle (TiO_2_-NP).

Previous studies using higher concentrations of nanoparticles than in the present study have shown that nanoparticle exposure during pregnancy causes malformations, behavioral changes, and alterations in neurotransmitter levels in the offspring (Jackson et al., 2011; Ghaderi et al., 2015; Notter et al., 2018; Umezawa et al., 2018; Zhang et al., 2019; Tarlan et al., 2020; Zhang et al., 2020); however, these alterations were not observed in the present low-dose exposure study. In addition, microglial activation was not observed in the brains of offspring maternally exposed to CB-NP, as described above (Figure 2). The evidence and our findings indicate that exposure to low-doses of nanoparticles, which does not induce significant effects on their phenotype, including behavioral function and neurotransmitter levels in the brain, can cause brain perivascular abnormalities such as PVM denaturation. Brain perivascular injuries may occur before abnormal neurotransmitter levels or behavioral abnormalities are detected as phenotypes because the perivascular tissues are very sensitive to foreign substances and stimuli. Thus, histological alteration of PVMs and their surrounding tissues may be a sensitive end-point to investigate adverse effects on the developing brain.

The potential mechanism by which PVMs and tissues around blood vessels in the brain are susceptible is that they function as a destination for waste products and foreign substances (like a garbage dumps) (Benveniste et al., 2019), but the mechanisms have not yet been clearly revealed. Further studies are needed to clarify why and how the brain perivascular tissues are sensitive to maternal exposure to foreign substances. In particular, the contents in the PAS-positive granules of PVMs were not identified. Bioimaging technology for high-resolution analysis are needed to clarify its contents. In addition, the present study provides no evidence of any relationship between PVM denaturation and abnormalities in brain function. Indeed, previous studies have suggested that PVM abnormalities are associated with pathological changes such as neuroinflammation and arterial hypertension and several disease processes including brain infections, neurodegenerative diseases, and neurovascular-neurocognitive dysfunction (Faraco et al., 2017). However, it is unclear how the PVM denaturation observed following the low-dose exposure to nanoparticle in the present study lead to changes in brain function. Therefore, further studies are needed to link the slight changes in PVMs to the resulting phenotype. On the other hand, there are still not many findings about PVMs. Since the function and localization of PVMs suggest that it is significant in protecting the brain tissue from accumulation of waste products and foreign substances and contribute to maintaining the brain at a normal state, investigation of brain perivascular areas including PVMs will contribute to a better understanding of developmental neurotoxicity.

Furthermore, we chose low-dose exposure to nanoparticles as a weak stimulus in the present study but did not investigate other toxicants. Therefore, it is necessary to evaluate whether PVMs will sensitively respond to other toxicants as well as nanoparticles. The PAS-immunohistochemical double-staining method indicated in the present study may be effective for the further investigation of the histological characteristics of brain perivascular tissues and effects of various substances on the tissues.

## Conclusion

In the present study, we established a novel staining method to sensitively determine the histopathological abnormalities around brain blood vessels. In particular, our investigation showed that MMR-positive PVMs with autofluorescence emission were identical to Mato cells stained by PAS staining. Taking advantage of PAS staining to detect mild denaturation of PVMs, a PAS-immunohistochemical double-staining method for brain tissue was developed. Furthermore, the present study determined the optimal procedures for the staining method and introduced their results. This double staining method enabled easy detection and rapid evaluation of brain perivascular abnormalities, including the denaturation of PVM granules.

Since PVMs with unique localization and phagocytic function are essential to protect the brain from adverse effects caused by waste products and foreign substances, PVMs and their surrounding tissues respond sensitively to even slight stimuli (Guillemin and Brew, 2004; Faraco et al., 2017; Abreu et al., 2019a). In fact, our findings also indicated that exposure to low-doses of nanoparticles, which does not affect microglial activation, behavioral function, and neurotransmitter levels in the brain, induced brain perivascular abnormalities including PVM denaturation. Thus, their histological changes may be a potential end-point for evaluating adverse effects on the developing brain. In the present study, the slight responses of brain perivascular tissues, such as mild denaturation of PVMs, were sensitively captured by the PAS-immunohistochemical double-staining method. Therefore, the histopathological evaluation of brain perivascular areas using this staining method may contribute to the sensitive detection and assessment of developmental neurotoxicity.

This double staining also allows evaluation of the histopathological denaturation of the PVMs and the associated abnormalities in the surrounding tissues in the same section. The present study demonstrated that enlargement of PAS-stained granules in PVMs was only detected near activated astrocytes that highly expressed GFAP. As in the present study, where the expression of GFAP and the morphology of PVM were combinedly analyzed in the same section, using this double staining method may accelerate the investigation of the relationship between the expression of various molecules and the morphology of PAS-positive PVMs. In addition, this staining method could also be used to evaluate meningeal macrophages, which plays the same role as PVMs at different localization (Goldmann et al., 2016; Faraco et al., 2017; Yang et al., 2019). This double staining method will provide new insights into the functional relationship between PVMs and the surrounding cells in terms of their localization.

## Data Availability

The original contributions presented in the study are included in the article/supplementary material, further inquiries can be directed to the corresponding author.

## Abbreviations

3-MT, 3-Methoxytyramine; 5-HIAA, 5-Hydroxyindole-3-acetic acid; 5-HT, Serotonin (5-Hydroxytryptamine); Ad, Adrenaline (Epinephrin); CB-NP, Carbon black nanoparticles; CEA, Central Nucleus of Amygdale; Cpu, Caudate-putamen/neostriatum; DA, Dopamine; DAB, 3,3′-Diaminobenzidene; DOPAC, 3,4-Dihydroxyphenylacetic acid; RN, Dorsal Raphe Nucleus; GFAP, Glial fibrillary acidic protein; HVA, Homovanillic acid; MHPG, 3-Methoxy-4-hydroxyphenylglycol; MMR, Macrophage mannose receptor; MRF, Medullary Reticular Formation; NA, noradrenaline (norepinephrine); NAcc, Nucleus Accumbens; NM, Normetanephrin; PFC, Prefrontal Cortex; PND, Postnatal day; PAS, Periodic acid-Schiff; PVM, Perivascular macrophage; PVN, Paraventricular Hypothalamic Nucleus; SD, Standard deviation; TiO_2_-NP, Titanium dioxide nanoparticle; VTA, Ventral Tegmental Area.
